# Association between Dairy Product Intake and Risk of Fracture among Adults: A Cohort Study from China Health and Nutrition Survey

**DOI:** 10.3390/nu14081632

**Published:** 2022-04-14

**Authors:** Xiaona Na, Yuandi Xi, Sicheng Qian, Jian Zhang, Yucheng Yang, Ai Zhao

**Affiliations:** 1 Vanke School of Public Health, Tsinghua University, Beijing 100084, China; nxn21@mails.tsinghua.edu.cn (X.N.); yyucheng@mail.tsinghua.edu.cn (Y.Y.); 2 Institute for Healthy China, Tsinghua University, Beijing 100084, China; 3 School of Public Health, Capital Medical University, Beijing 100069, China; xiaoer711@163.com; 4 Faculty of Science, McGill University, Montreal, QC H3A 0G4, Canada; sicheng.qian@mail.mcgill.ca; 5 School of Public Health, Peking University, Beijing 100091, China; zhangjian92@pku.edu.cn

**Keywords:** dairy products, fracture, mediation effects, Chinese adults

## Abstract

Background: The current literature reports inconsistent associations between dairy product intake and fracture. This study assessed the association between dairy product intake and the risk of fracture among Chinese adults and examined the mediation effects of height and body mass index (BMI) on the association. Methods: Data in 1997–2015 from the China Health and Nutrition Survey were used. Dietary data were collected by a 24-hour dietary recall, and occurrences of fracture were obtained by self-report of participants. Cumulative average intake of daily dairy products was calculated by the sum of the dairy product intake and divided by the total waves of participating in the surveys before fracture. Cox proportion hazard regressions were performed to explore the associations between dairy product intake and the risk of fracture. Mediation analysis models were established to examine the mediation effects of height and BMI on the associations. Results: A total of 14,711 participants were included. Dairy product intake of 0.1–100 g/day was associated with a decreased risk of fracture, while no association was observed among participants with dairy product intake of >100 g/day. The indirect effects of dairy product intake on the fracture mediated by height and BMI were much smaller than the direct effects. Conclusions: Dairy product intake with 0.1–100 g/day is associated with a lower risk of fracture, and the association is mainly a direct result of nutrients in dairy products and much less a result of the mediation effects of height or BMI. Dairy product intake of 0.1–100 g/day might be a cost-effective measure for Chinese adults to decrease fracture incidence.

## 1. Introduction

Fracture is a major global public health concern and causes numerous disabilities and mortalities around the world. The global number of fractures that occurred in 2019 was estimated to be 178 (95% confidence interval (CI): 162–196) million, increased by 33.4% from 1990 [1]. Particularly in China, the fracture incidence was projected to increase from 7.15/1000 people in 2010 to 9.84/1000 people in 2050 among people aged at least 50 years, with costs increasing from USD 9.45 billion to USD 25.43 billion (the costs were converted to 2013 USD) [2]. It is estimated that 40.9% and 8.7% of Chinese women and men aged 50 years and above, respectively, encountered osteoporotic fractures in their remaining lifetime [3].

Dairy products provide energy and multiple nutrients for human bone health, such as calcium and protein [4], and are listed in dietary guidelines in many countries [5,6,7]. Dairy products have been found to be associated with a lower risk of fracture, diabetes, and mortality in previous studies [8,9]. However, inconsistent health effects of dairy products were reported in recent research [10,11,12,13,14]. Several systematic reviews and meta-analyses concluded that dairy product intake was not related to fracture [12,13,15,16]. The conclusions of cohort studies are also contradictory [10,13,14,17,18,19], apart from no relations observed in some research, some cohort studies provided evidence that higher dairy product intake increased the risk of fracture in western countries [10,18]. The association between dairy products and the fracture was inferred to be mediated by height by some research, that is, dairy products contributed to growing tall, while higher height further increased the risk of fracture [20,21]. However, the mediation of height on the association between dairy products and the risk of fracture has not been completely verified.

A large prospective study showed that the median (inter quartile range (IQR)) dairy product intake was 20.5 (5.17–75.0) g/day among Chinese adults aged 45 to 74 years during 1999–2010 [22]. Dairy product intake is much lower in China than in America and European countries and is below the recommendation of the Chinese Dietary Guideline in 2016 for adults (300 g/day) [6,23], which causes an insufficient intake of multiple nutrients, such as calcium and protein [22]. Previous studies showed that the effect of dairy products on health partially depended on the diet quality of people. Based on different intake statuses of nutrients, such as calcium and protein, between populations in China and other countries [20], we hypothesized that the effect of dairy products on bone health in China may be very different from that in countries. Nevertheless, the effect of dairy products on fracture among Chinese adults and the mediation of height and BMI on the effect is unknown.

To address these gaps, this study aimed to investigate: (1) the effect of dairy product intake on fracture among Chinese adults, and (2) the mediation effect of height and BMI on the association between dairy product intake and the risk of fracture.

## 2. Materials and Methods

### 2.1. Study Population

Data used in this study were from the China Health and Nutrition Survey (CHNS). CHNS is an ongoing longitudinal survey initiated in 1989 in China, and nine additional waves were followed in 1991, 1993, 1997, 2000, 2002, 2004, 2006, 2009, 2011, and 2015 (dietary data in 2015 were not available). Multi-stage random cluster sampling was conducted in 11 provinces and four autonomous cities or districts to obtain the final samples. More detailed information about the survey was shown on the study website [24].

**Figure 1:**
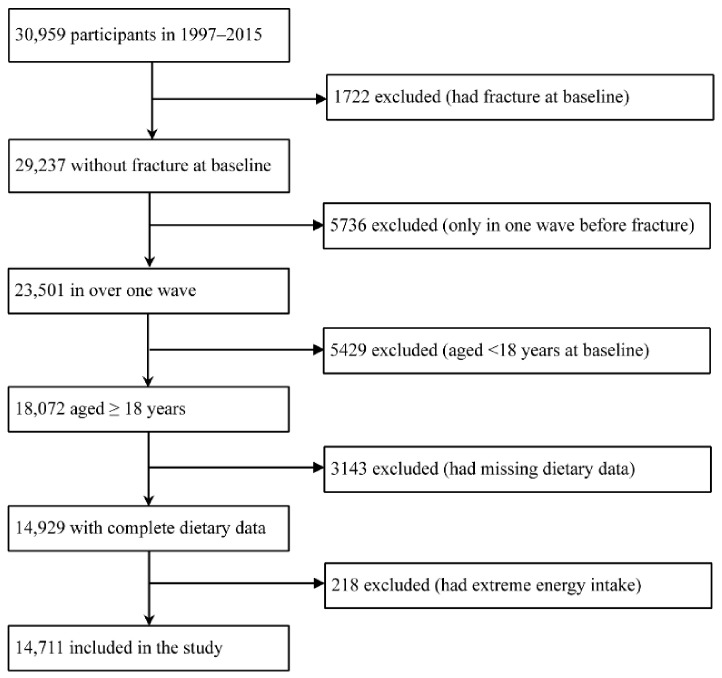
Flow chart of included participants.

In our study, data from 1997 to 2015 were selected to investigate the association between dairy product intake and the risk of fracture, because dietary data in 1991 and 1993 were not correctly coded and could not be identified. Participants were excluded if they met one of the following conditions: (1) had a fracture at baseline, (2) only in one wave before the occurrence of fracture, (3) aged < 18 years at baseline, (4) had missing dietary or fracture data, or (5) had extreme daily total energy intake (<500 kcal/day or >8000 kcal/day). A final of 14,711 participants were included in our study (Figure 1).

Calculation of the minimum sample size was according to Formula 1, with *p*_1_ and *p*_0_ referring to fracture incidence in exposure and control groups, respectively. $\bar{p}$ was the mean value of *p*_1_ and *p*_0_, and *q*_1_, *q*_0_, and $\bar{q}$ were equal to 1 − *p*_1_, 1 − *p*_0_, and 1 − $\bar{p}$, respectively. In this study, type I error *α* = 0.05, type Ⅱ error *β* = 0.10, *p*_0_ = 0.013 (estimated by a global representative survey in 2019) [1], *p*_1_ = relative risk (RR) × *p*_0_ = 0.67 × 0.013 = 0.009 (RR was estimated by a randomized control trial exploring the effects of dairy products on the fracture incidence) [25]. With these parameters, the sample size was estimated to be 9557, and the number of our included participants satisfied the demand of the minimum sample size.

(1) $$n=\frac{{\left(Z_{1-\alpha /2}\sqrt{2\bar{pq}}+Z_{\beta }\sqrt{p_{0}q_{0}+p_{1}q_{1}}\right)}^{2}}{{\left(p_{1}-p_{0}\right)}^{2}}$$

### 2.2. Dietary Assessment

Dietary intake information was collected at individual and household levels. Dietary intake at the individual level was collected by trained interviewers in three consecutive days selected randomly within a week. Types, quantities of all foods consumed, and the places consumed over the past 24 h were reported by participants with the help of food models and standardized food pictures, and then recorded and coded by the interviewers. Purchased, homemade, and processed food and condiments at the household level were weighed and recorded at the beginning and the end of the three-day survey to calculate their consumption.

In our study, dietary data at the individual level was used to calculate the food and nutrient intakes. Dairy products in our study were identified as liquid cow’s milk, yogurt, milk powder, and cheese. Then, 100 g yogurt, 12.5 g milk powder, and 10 g cheese were converted to 100 g liquid cow’s milk according to their protein ratios with fresh milk in light of the Chinese Dietary Guideline in 2016 [6]. The cumulative average intake of daily dairy products, for the participants never suffering from a fracture, was calculated by the sum of dairy product intake divided by the total waves of participating in the surveys. For those suffering from fractures, the cumulative average intake of daily dairy products before suffering from a fracture for the first time was calculated. Dairy product intake was categorized into three groups: no consumption, 0.1–100 g/day, and >100 g/day. Since the food coding method was changed in the dietary survey after 2000, the nutrients (including calcium, protein, and phosphorus) and total energy intake from 1997 to 2000 and after 2000 were calculated based on China Food Composition and China Food Composition Table 2002/2004, respectively [26,27,28].

### 2.3. Definition of Fracture

Whether occurred, age at the first occurrence, and times of fracture were self-reported by participants in each wave. In our study, information for the first record of fracture was kept to reduce the recall bias, and no fracture occurred was defined as that a participant did not report any fractures in all the waves.

### 2.4. Definition of Covariates

Participants should complete questionnaires containing the following information: (1) demographic characteristics, including age (years), sex (male and female), survey wave (1997–2015), educational level (junior high school or below and senior high school or above), region of residence (eastern, central, and western region), place of residence (urban and rural area), and individual annual income (yuan); (2) lifestyles, including smoking history (no and yes), drinking history (no and yes), and physical activity. The physical activity level of each participant was quantified by multiplying the intensity (metabolic equivalent of task, MET), frequency (weekly), and duration (hour) of physical activity, with MET-hour/week as the unit. MET of each specific physical activity type was obtained according to the Compendium of Physical Activities [29], which is an objective measurement of energy expenditure; (3) chronic disease history (no and yes), including hypertension, diabetes, cancer, and stroke. In addition, weight and height were measured by trained surveyors with standard measurement methods and instruments. Body mass index (BMI) was calculated by dividing weight (kg) by the square of height (m^2^).

### 2.5. Statistical Analyses

All statistical analyses were performed by R 4.1.1 (R Development Core Team, Vienna, Austria). *p*-value < 0.05 (two-tailed) was considered statistically significant.

All the analyses were stratified by sex. Chi-square (χ^2^) tests (for categorical variables), one-way ANOVA (for continuous variables that satisfied criteria of the normal distribution and homoscedasticity), and Kruskal–Wallis test (for continuous variables that did not satisfy criteria of the normal distribution or homoscedasticity) were used to compare statistical differences of demographic characteristics, lifestyles, and health status across different dairy product intake groups. The association between dairy products and the risk of fracture was explored by Cox proportion hazard regression models, including an unadjusted model and two adjusted models. Model 1 adjusted for age, sex, educational level, region of residence, educational level, individual annual income, smoking history, drinking history, BMI, chronic disease history, and physical activity. Model 2, based on Model 1, further adjusted for intakes of total energy and total calcium.

Intakes of calcium and protein in dairy products were categorized into three groups: no, moderate, and high consumption, where the cut-off values of moderate and high consumption were calculated by their median intake in dairy products. To further investigate the association of calcium and protein in dairy products with the risk of fracture, Cox proportional hazard regression models of the two nutrients were established. For models of calcium in dairy products, Model 1 adjusted for age, sex, educational level, region of residence, individual annual income, smoking history, drinking history, BMI, chronic disease history, and physical activity. Model 2, based on Model 1, further adjusted for total energy intake. Dietary calcium/phosphorus intake ratio was associated with bone mass [30,31], so Model 3, based on Model 2, further adjusted for total phosphorus intake. The adjusted variables of Model 1 and Model 2 in Cox regression models of protein in dairy products were the same as those of calcium, except that Model 3 adjusted for total intakes of calcium and phosphorus.

To investigate the nonlinear association between dairy product intake and the risk of fracture, restricted cubic spine Cox regressions were performed among overall participants and subgroups for men, women, and women aged ≥ 45 years, adjusting for the same confounding variables as model 2 of Cox regression models of dairy products. The number of knots with max *R*^2^ and *Dxy* in the restricted cubic spine Cox regression model was selected. In addition, mediation analysis models using R package *lavaan* (0.6-11 version) were established to examine the mediation effects of height and BMI on the association between dairy product intake and the risk of fracture among overall participants and subgroups by setting two dummy variables of dairy product intake to enter the mediation analysis models.

## 3. Results

### 3.1. Basic Characteristics of Participants

A total of 14,711 participants (6884 men and 7827 women) were included in this study. The median (*IQR*) age at baseline was 42.0 (32.0, 54.0) years. Total of 11,768 (80.0%) participants did not consume dairy products, 2041 (13.9%) and 902 (6.1%) consumed 0.1–100 g/day and >100 g/day, respectively.

Table 1 shows the sociodemographic characteristics, lifestyles, and health status across different dairy product intake groups among overall, male, and female participants. Compared to the no consumption group, dairy product intake of 0.1–1 g/day and >100 g/day groups tended to be older, female, less educated, and physically active and inclined to live in eastern regions and have higher individual annual income. The proportion of smoking history was slightly lower, but the proportion of chronic disease history was slightly higher among participants who consumed dairy products of 0.1–1 g/day and >100 g/day than those who consumed no dairy products. The intakes of total calcium and calcium in dairy products were much higher among participants who consumed dairy products of 0.1–1 g/day and >100 g/day compared to the no consumption group.

### 3.2. Association between Dairy Product Intake and the Risk of Fracture

Table 2 shows the association between dairy product intake and the risk of fracture. During a median (*IQR*) follow-up of 9.0 (4.0, 15.0) years with 147,709.0 person-years, 505 fractures occurred. Incidences of fracture among participants with average dairy product intake of 0 g/day, 0.1–100 g/day, and >100 g/day were 3.750/1000, 1.904/1000, and 1.861/1000 person-years, respectively. In unadjusted and adjusted Cox regression models, for the overall participants, dairy product intake of 0.1–100 g/day was associated with a lower risk of fracture compared to no consumption group (hazard ratio (HR): 0.49, 95% CI: 0.34–0.70), while no association was observed among the participants with dairy product intake of >100 g/day (HR: 0.65, 95% CI: 0.33–1.32). The results of sex-stratified analyses were similar to overall participants: only significant associations between dairy product intake of 0.1–100 g/day and the risk of fracture were observed after full adjustments for both men and women.

### 3.3. Associations of Calcium and Protein in Dairy Products with the Risk of Fracture

Results of the relationships of calcium and protein in dairy products with the risk of fracture are shown in Table 3 and Table 4. After full adjustments, increasing intakes of calcium and protein in dairy products were associated with a decreased risk of fracture compared to the no consumption group for overall participants and also for men and women.

### 3.4. Nonlinear Associations between Dairy Product Intake and the Risk of Fracture

**Figure 2:**
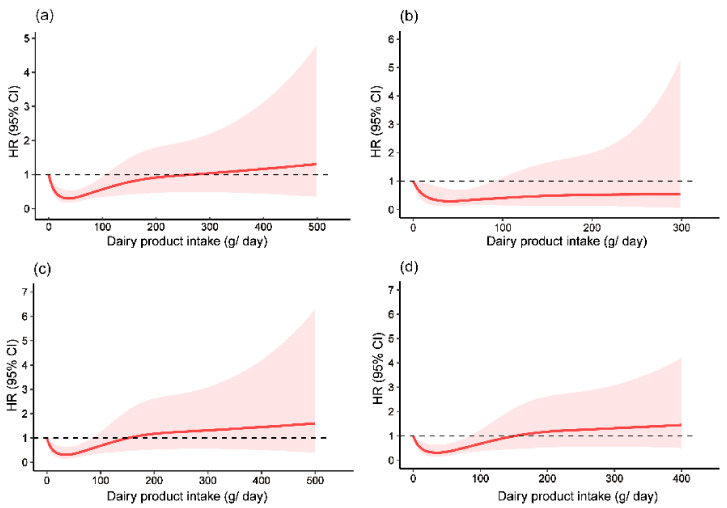
Restricted cubic spine plots to evaluate relationships between dairy product intake and fracture among (**a**) overall participants; (**b**) men; (**c**) women; and (**d**) women aged ≥ 45 years. HR and 95% CI were adjusted for age, sex, education level, region of residence, individual annual income, smoking history, drinking history, physical activity, BMI, chronic disease history, and intakes of total energy and total calcium.

Figure 2 shows the nonlinear relationships between dairy product intake and the risk of fracture among the overall participants and subgroups for men, women, and women aged ≥ 45 years. RCS models with four knots were selected because of max *R*^2^ and *Dxy* in the models. Among the overall participants and all the subgroups, dairy product intake of 0.1–100 g/day was associated with a decreased risk of fracture, whereas >100 g/day was not associated with fracture.

### 3.5. Mediation Effects of Height and BMI on the Associations between Dairy Product Intake and the Risk of Fracture

**Figure 3:**
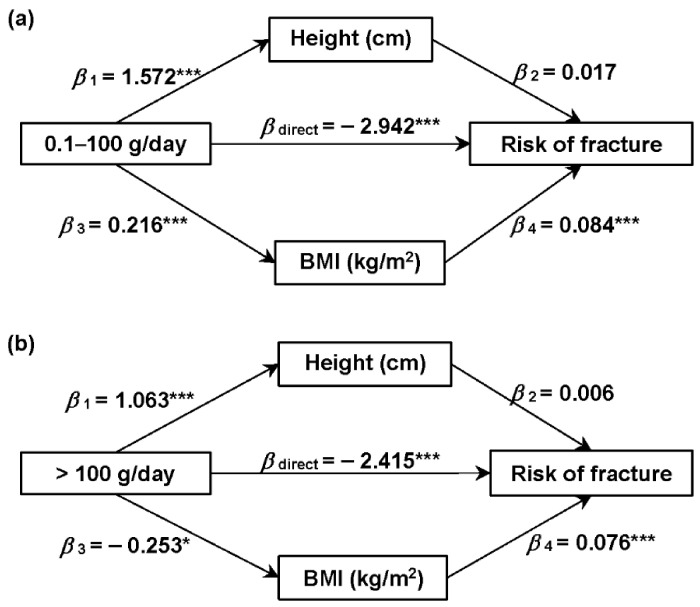
Mediation effect of height and BMI on the association of dairy product intake of (**a**) 0.1–100 g/day and (**b**) >100 g/day with the risk of fracture compared to no consumption of dairy products. Data are regression coefficients with adjustment for covariates (including age, sex, educational level, region of residence, individual annual income, smoking history, drinking history, chronic disease history, physical activity, and intakes of total energy and total calcium). * *p* < 0.05, *** *p* < 0.001.

Figure 3 shows the mediation effects of height and BMI on the associations between dairy product intake and the risk of fracture. The direct effect of dairy product intake of 0.1–100 g/day on the risk of fracture was estimated at −2.942 (*p* < 0.001), and the indirect effect was estimated at 0.018 caused by the increased BMI (*β*_diary-BMI_ = 0.216, *p* < 0.01; *β*_BMI-fracture_ = 0.084, *p* < 0.001). The direct effect of dairy product intake of >100 g/day on the risk of fracture was estimated at −2.415 (*p* < 0.001), and the indirect effect was estimated at −0.019 caused by the reduced BMI (*β*_diary-BMI_ = −0.253, *p* < 0.05; *β*_BMI-fracture_ = 0.076, *p* < 0.001). The direct effects in subgroup analyses were similar to the overall participants, but the mediation effects of height and BMI were different. The associations were positively mediated by increased height among men, but were not mediated by height or BMI among women. In women aged ≥ 45 years, the association of dairy product intake of 0.1–100 g/day with fracture incidence was mediated by increased BMI (Supplementary Figure S1).

For the mediation effect of height, dairy could increase the height among all the subgroups, yet the increased risk of fracture with the increased height could only be found among men. For BMI, dairy product intake with 0.1–100 g/day might increase the BMI among overall participants and women, but BMI was positively associated with the risk of fracture for men and women aged ≥ 45 years. In contrast, dairy product intake with >100 g/day might reduce BMI among overall participants, but did not in subgroups, and BMI was positively associated with the risk of fracture for men and women aged ≥ 45 years (Supplementary Figure S1).

## 4. Discussion

Currently, the effects of dairy products on health have aroused extensive concern. In this national prospective study, we first explored the association between dairy product intake and the risk of fracture among Chinese adults and examined the mediation effects of height and BMI on the association. We found that dairy product intake of 0.1–100 g/day was negatively associated with the risk of fracture among men and women, and the association was mainly a direct result of nutrients in dairy products and much less the result of the mediation of height or BMI. The calcium and protein in dairy products may explain the negative association between dairy products and fracture incidence.

### 4.1. Association between Dairy Product Intake and the Risk of Fracture

The health effects of dairy products/milk have been debated for years, but epidemiological literature on dairy product intake and the risk of fracture is still limited, especially among Chinese adults [10,13,14,17,18,19,32,33,34,35,36,37]. Our results support the conclusions of several cohort studies that dairy product intake was associated with a decreased risk of fracture among adults [14,17,34,37]. Feskanich and colleagues observed dairy product intake was associated with a significant 6% lower risk of hip fracture per daily serving (about 237 mL) among US adults over 50 years old (RR: 0.94, 95% CI: 0.90–0.98) [32]. In addition, one recent study with a randomized controlled trial design reported that dairy products with high calcium and protein could contribute to a 33% (HR: 0.67, 95% CI: 0.48–0.93) and 46% (HR: 0.54, 95% CI: 0.35–0.83) reduction of fractures and hip fractures, respectively, for Australian elder adults in two years [25]. This study provides stronger epidemiologic evidence for the protective effects of dairy products on fracture and supports our findings. Meanwhile, other cohort studies failed to examine this association [13,19,32,35,36]. For example, Benetou and colleagues showed dairy products did not appear to play a role in preventing hip fractures in a prospective study with 29,122 participants aged 60 years and above from five European countries [19]. Studies using data from the Framingham cohort found that dairy product intake was not related to the risk of hip fracture among both middle-aged and elderly populations, but positively related to bone mineral density [33,36]. Furthermore, a study containing two large-scale cohorts found that higher milk intake was associated with higher mortality for both Swedish women and men and associated with higher fracture incidence for Swedish women [10]. The potential harm of higher intake of dairy products on fracture could be explained by the evidence that milk is the main dietary source of D-galactose, which was proved to cause oxidative stress damage, chronic inflammation, neurodegeneration, and decreased immune response in animal experiments [38,39,40]. Chronic inflammation might increase osteoclast formation and finally lead to bone loss. The increase of oxidative stress damage might inhibit bone formation, break bone remodeling balance, decrease bone mineral density, and thereby cause age-related bone loss, sarcopenia, and fracture [38,39,40]. Based on the observation in the animal model of aging, it is estimated that 6–10 g of D-galactose (about correspond to 1–2 glasses of milk) might accelerate human senescence [10]. It should be noted that the above estimated adverse effect level is much higher than daily dairy product intake among Chinese adults (about 26.47 g/day in 2011) [41].

The contradictory conclusions of the studies might be explained by the fact that the effects of dairy products on bone health or other aspects of health partially depend on the quality of the diet without dairy products [20,42]. Although the prevalence of undernourishment in China has been greatly reduced in the past decades (from 23.9% in 1990–1992 to 9.3% in 2014–2016), it is still higher than in some other countries, such as Korea and America [43]. In addition, the insufficient intake of micronutrients is still common in China. For example, a study showed that the average dietary calcium intake was under 400 mg/day among Chinese adults, which was far less than that in other countries [44]. The benefit of dairy product intake might be especially obvious in insufficient calcium intake countries, while this benefit in countries where the calcium intake is sufficient might be compromised [42].

### 4.2. The Associations of Calcium and Protein in Dairy Products with the Risk of Fracture

To further explore the mechanism of the protective effect of dairy products, we additionally examined the associations between calcium and protein in dairy products and the risk of fracture. The results showed that calcium and protein in dairy products were negatively associated with the risk of fracture. Dairy products provide adequate calcium and high biological value protein for most populations [4,45]. Increased dietary calcium intake may suppress bone resorption to improve bone balance and prevent PTH-mediated bone loss, and thereby prevent fracture [4,45,46]. Protein in dairy products might benefit bone health by promoting calcium absorption, stimulating the secretion of insulin-like growth factor-1, and enhancing lean body mass [47,48]. Furthermore, other nutrients in dairy products also play important roles to maintain bone health [45].

### 4.3. The Appropriate Dairy Product Intake for Chinses Adults

Since the effects of dairy products on health vary among different populations, the most appropriate dairy product intake amount for certain populations is required to consider with caution. The results of RCS Cox regression suggest that dairy product intake of 0.1–100 g/day might provide higher health effects to prevent fractures among Chinese adults. However, no significant associations were observed between dairy product intake of >100 g/day and the risk of fracture, which might be explained by a specific threshold level of calcium balance for the Chinese population. Previous studies showed that calcium balance threshold levels were about 1000 mg/day and 400–500 mg/day among American adults and Chinese adults, respectively [49,50]. Considering synthetically our results and other research [15,51,52], dairy product consumption of 0.1–200 g/day on health was optimal and beneficial, which is slightly lower than the current recommendation for the Chinese adults (300 g/day) [6].

### 4.4. The Mediation Effects of Height and BMI on the Associations between Dairy Products and the Risk of Fracture

Previous studies inferred that height might mediate the association of dairy products with the risk of fracture [53]. In our study, we explored the mediation effects of height and BMI on this association to explore the possible mechanisms of dairy products on bone health among Chinese adults. In our results, apart from the negative direct association between dairy product intake and the risk of fracture, we found that the mediation effects of height and BMI on the associations were very limited and different among subgroups. For the mediation effect of height, interestingly, dairy could increase the height among all the subgroups, while the increased risk of fracture with the increased height could only be found among men, which is in line with the previous study [53]. For BMI, dairy product intake of 0.1–100 g/day might increase the BMI among women, but BMI was positively associated with the risk of fracture for men and women aged ≥ 45 years. Oppositely, dairy product intake of >100 g/day might reduce the BMI among overall participants but did not in subgroups, and BMI was positively associated with the risk of fracture for men and women aged ≥ 45 years, which might result from that high BMI was associated with unhealthy body and further increased the risk of fracture [54].

Our results showed an oppositely direct and indirect association between dairy product intake and fracture incidence, but the direct association was apparently higher than the indirect association. Therefore, we infer the negative association between dairy products and fracture might be caused by calcium and protein in them. In addition, the sex and age heterogeneity of mediation effects of height and BMI were found in the subgroup analyses. Although substantial studies have examined the relationships of dairy product intake with height, weight, and BMI [55,56,57] and the relationships of height, weight, and BMI with the risk of fracture [21,54,58,59,60,61], their contradictory results could not draw a conclusion. Therefore, the specific mechanisms of mediation effects of height, weight, and BMI on the association are complex and need further experiments and epidemiological studies to verify.

### 4.5. Strengths and Limitations

The strengths of our study include a prospective design with a large sample size and a good representation of Chinese adults. Moreover, it is the first study to explore the association between dairy product intake and the risk of fracture and examine whether the association is mediated by height or BMI among Chinese adults. However, some limitations still exist in our study and should be taken with caution. Firstly, the types and fat contents of dairy products were not considered in the study design, while different types of dairy products and fat contents may have various health effects [62,63,64]. Secondly, only calcium and protein in dairy products were taken into account in our study, and the effect of nutrients and bio-actives in dairy products as a matrix on bone health should also be investigated in future research. Thirdly, dairy product intake was grouped into three groups because of the limited number of participants who consumed dairy products, so the health effects of dairy product intake of >100 g/day might be misevaluated. Fourthly, the fracture was based on self-report and the specific site of fracture was not been investigated, so some cases might be missed. Fifthly, measurement bias cannot be eliminated, which is unavoidable for all epidemiological studies, especially for the dietary survey. Finally, the causality of the association could not be established in this observational study, although it was based on a prospective cohort.

## 5. Conclusions

In the context of the contradictory conclusions regarding bone health effects of dairy products, different nutritional statuses around the world, and limited relevant studies in China, we first investigate the association between dairy product intake and the risk of fracture. The results of this nation-representative and prospective study suggest that dairy product intake of 0.1–100 g/day is associated with a lower risk of fracture. In addition, the association is mainly a direct result of nutrients in dairy products and much less a result of the mediation effects of height or BMI. From a public health point of view, dairy product intake of 0.1–100 g/day with an effect on fracture incidence might be a cost-effective measure for Chinese adults. Further randomized controlled trials need to be conducted to verify our conclusions and determine the optimal amount of dairy product intake among Chinese adults.

## Figures and Tables

**Table 1:** Basic demographic, lifestyle, and dietary intake characteristics across dairy product intake groups in overall, male, and female participants.

Overall Participants					
		No Consumption	0.1–100 g/Day	>100 g/Day	*p*
		(*n* = 11,768)	(*n* = 2041)	(*n* = 902)	
Age at baseline (years)		41.0 (31.0, 53.0)	43.0 (33.0, 55.0)	51.0 (38.0, 61.0)	<0.001
Sex					0.001
	Men	5595 (81.3)	911 (13.2)	378 (5.49)	
	Women	6173 (78.9)	1130 (14.4)	524 (6.69)	
Education level					<0.001
	Junior high school or below	2455 (61.6)	976 (24.5)	557 (14.0)	
	Senior high school or above	9313 (86.9)	1065 (9.93)	345 (3.22)	
Region of residence					<0.001
	Eastern region	3820 (68.0)	1086 (19.3)	710 (12.6)	
	Central region	4693 (86.1)	688 (12.6)	69 (1.27)	
	Western region	3255 (89.3)	267 (7.33)	123 (3.37)	
Place of residence					<0.001
	Urban area	3398 (61.5)	1386 (25.1)	742 (13.4)	
	Rural area	8370 (91.1)	655 (7.13)	160 (1.74)	
Individual annual income (yuan)		6347 (2721, 13,137)	10,131 (5738, 18,000)	21,000 (12,973, 31,858)	<0.001
Smoking history					<0.001
	No	7994 (78.8)	1446 (14.2)	708 (6.98)	
	Yes	3773 (82.7)	594 (13.0)	193 (4.23)	
Drinking history					<0.001
	No	7689 (80.5)	1248 (13.1)	611 (6.40)	
	Yes	4079 (79.0)	793 (15.4)	291 (5.64)	
BMI (kg/m^2^)		22.8 (20.9, 25.1)	23.4 (21.4, 25.7)	23.5 (21.4, 25.8)	<0.001
Chronic disease history					<0.001
	No	8752 (81.6)	1374 (12.8)	603 (5.62)	
	Yes	3016 (75.7)	667 (16.8)	299 (7.51)	
Physical activity (MET-hour/week)		132 (0.00, 614)	180 (11.6, 605)	501 (117, 1266)	<0.001
Intake of total energy (kcal/day)		2094 (1766, 2437)	2047 (1754, 2333)	1885 (1554, 2217)	<0.001
Intake of total calcium (mg/day)		342 (271, 432)	411 (337, 503)	584 (471, 732)	<0.001
Intake of calcium in dairy products (mg/day)		0.00 (0.00, 0.00)	39.3 (15.7, 69.3)	173 (139, 236)	<0.001
Men					
		**No consumption**	**0.1–100 g/Day**	**>100 g/Day**	***p***
		**(*n* = 5595)**	**(*n* = 911)**	**(*n* = 378)**	
Age at baseline (years)		42.0 (31.0, 53.0)	44.0 (34.0, 55.0)	54.0 (39.0, 64.0)	<0.001
Education level					<0.001
	Junior high school or below	1407 (65.9)	471 (22.1)	256 (12.0)	
	Senior high school or above	4188 (88.2)	440 (9.26)	122 (2.57)	
Region of residence					<0.001
	Eastern region	1832 (69.7)	489 (18.6)	307 (11.7)	
	Central region	2221 (86.9)	305 (11.9)	29 (1.14)	
	Western region	1542 (90.7)	117 (6.88)	42 (2.47)	
Place of residence					<0.001
	Urban area	1630 (63.5)	619 (24.1)	316 (12.3)	
	Rural area	3965 (91.8)	292 (6.76)	62 (1.44)	
Individual annual income (yuan)			11,866 (6770, 20,281)	24,000 (14,118, 36,917)	<0.001
Smoking history					<0.001
	No	2048 (78.3)	365 (14.0)	203 (7.76)	
	Yes	3547 (83.1)	545 (12.8)	175 (4.10)	
Drinking history					<0.001
	No	2086 (82.2)	284 (11.2)	168 (6.62)	
	Yes	3509 (80.7)	627 (14.4)	210 (4.83)	
BMI (kg/m2)			23.9 (21.7, 25.8)	23.9 (22.0, 26.0)	<0.001
Chronic disease history					<0.001
	No	4152 (83.4)	590 (11.9)	235 (4.72)	
	Yes	1443 (75.7)	321 (16.8)	143 (7.50)	
Physical activity (MET-hour/week)		7.70 (0.00, 282)	40.8 (0.00, 347)	274 (23.4, 1054)	<0.001
Intake of total energy (kcal/day)		2299 (1968, 2628)	2245 (1955, 2535)	2069 (1746, 2405)	<0.001
Intake of total calcium (mg/day)		363 (290, 458)	439 (359, 531)	606 (501, 751)	<0.001
Intake of calcium in dairy products (mg/day)		0.00 (0.00, 0.00)	38.7 (14.7, 69.3)	173 (136, 235)	<0.001
Women					
		**No consumption**	**0.1–100 g/Day**	**>100 g/Day**	***p***
		**(*n* = 6173)**	**(*n* = 1130)**	**(*n* = 524)**	
Age at baseline (years)		41.0 (30.0, 53.0)	43.0 (32.0, 54.0)	48.0 (37.0, 60.0)	<0.001
Education level					<0.001
	Junior high school or below	1048 (56.5)	505 (27.2)	301 (16.2)	
	Senior high school or above	5125 (85.8)	625 (10.5)	223 (3.73)	
Region of residence					<0.001
	Eastern region	1988 (66.5)	597 (20.0)	403 (13.5)	
	Central region	2472 (85.4)	383 (13.2)	40 (1.38)	
	Western region	1713 (88.1)	150 (7.72)	81 (4.17)	
Place of residence					
	Urban area	1768 (59.7)	767 (25.9)	426 (14.4)	
	Rural area	4405 (90.5)	363 (7.46)	98 (2.01)	
Individual annual income (yuan)		5455 (2327, 11,121)	8955 (5000, 15,526)	19,000 (11,549, 28,859)	<0.001
Smoking history					0.51
	No	5946 (78.9)	1081 (14.4)	505 (6.70)	
	Yes	226 (77.1)	49 (16.7)	18 (6.14)	
Drinking history					<0.001
	No	5603 (79.9)	964 (13.8)	443 (6.32)	
	Yes	570 (69.8)	166 (20.3)	81 (9.91)	
BMI (kg/m^2^)		22.8 (20.9, 25.2)	23.2 (21.2, 25.6)	23.0 (21.0, 25.5)	0.007
Chronic disease history					<0.001
	No	4600 (80.0)	784 (13.6)	368 (6.40)	
	Yes	1573 (75.8)	346 (16.7)	156 (7.52)	
Physical activity (Met-hour/week)		222 (101, 917)	233 (126, 750)	591 (204, 1398)	<0.001
Intake of total energy (kcal/day)		1937 (1651, 2223)	1885 (1652, 2146)	1769 (1462, 2075)	<0.001
Intake of total calcium (mg/day)		323 (258, 408)	392 (320, 475)	551 (459, 723)	<0.001
Intake of calcium in dairy products (mg/day)		0.00 (0.00, 0.00)	39.6 (17.3, 69.3)	173 (139, 236)	<0.001

**Table 2:** Association between dairy product intake and the risk of fracture.

	No Consumption	0.1–100 g/Day	>100 g/Day
	(*n* = 11,768)	(*n* = 2041)	(*n* = 902)
Overall Participants			
Incidence (no. of fractures/ 1000 person-years)	3.750	1.904	1.861
Unadjusted Model	1.00 (Reference)	0.50 (0.36, 0.69) ***	0.54 (0.29, 1.02)
Model 1	1.00 (Reference)	0.38 (0.27, 0.53) ***	0.32 (0.16, 0.64) **
Model 2	1.00 (Reference)	0.49 (0.34, 0.70) ***	0.65 (0.33, 1.32)
Men			
Incidence (no. of fractures/ 1000 person-years)	3.743	1.045	0.866
Unadjusted Model	1.00 (Reference)	0.27 (0.14, 0.52) ***	0.24 (0.06, 0.97) *
Model 1	1.00 (Reference)	0.23 (0.12, 0.45) ***	0.17 (0.04, 0.72) *
Model 2	1.00 (Reference)	0.32 (0.17, 0.62) **	0.36 (0.09, 1.50)
Women			
Incidence (no. of fractures/ 1000 person-years)	3.757	2.622	2.610
Unadjusted Model	1.00 (Reference)	0.69 (0.47, 1.01)	0.79 (0.39, 1.61)
Model 1	1.00 (Reference)	0.50 (0.33, 0.76) **	0.44 (0.20, 0.96) *
Model 2	1.00 (Reference)	0.62 (0.40, 0.96) *	0.80 (0.35, 1.83)

Model 1 adjusted for age, sex, educational level, region of residence, individual annual income, smoking history, alcohol status, BMI, chronic disease history, and physical activity. Model 2, based on Model 1, further adjusted for intakes of total energy and total calcium. * *p* < 0.05, ** *p* < 0.01, *** *p* < 0.001.

**Table 3:** Associations between calcium in dairy product intake and the risk of fracture.

	No Consumption	0.1–70 mg/Day	>70 mg/Day
	(*n* = 11,777)	(*n* = 1426)	(*n* = 1508)
Overall Participants			
Incidence (no. of fractures/ 1000 person-years)	3.747	1.892	1.922
Unadjusted Model	1.00 (Reference)	0.49 (0.34, 0.71) ***	0.54 (0.34, 0.86) *
Model 1	1.00 (Reference)	0.38 (0.26, 0.56) ***	0.35 (0.21, 0.58) ***
Model 2	1.00 (Reference)	0.39 (0.26, 0.58) ***	0.35 (0.21, 0.59) ***
Model 3	1.00 (Reference)	0.39 (0.27, 0.58) ***	0.37 (0.22, 0.61) ***
Men			
Incidence (no. of fractures/ 1000 person-years)	3.740	1.078	0.909
Unadjusted Model	1.00 (Reference)	0.28 (0.14, 0.57) ***	0.25 (0.09, 0.67) **
Model 1	1.00 (Reference)	0.24 (0.12, 0.50) ***	0.19 (0.07, 0.52) **
Model 2	1.00 (Reference)	0.25 (0.12, 0.51) ***	0.19 (0.07, 0.53) **
Model 3	1.00 (Reference)	0.25 (0.12, 0.52) ***	0.21 (0.07, 0.57) **
Women			
Incidence (no. of fractures/ 1000 person-years)	3.753	2.567	2.734
Unadjusted Model	1.00 (Reference)	0.67 (0.43, 1.02)	0.81 (0.48, 1.37)
Model 1	1.00 (Reference)	0.50 (0.32, 0.80) **	0.47 (0.26, 0.85) *
Model 2	1.00 (Reference)	0.52 (0.32, 0.82) **	0.47 (0.26, 0.86) *
Model 3	1.00 (Reference)	0.52 (0.32, 0.82) **	0.48 (0.27, 0.88) *

Model 1 adjusted for age, sex, educational level, region of residence, individual annual income, smoking history, drinking history, BMI, chronic disease history, and physical activity. Model 2, based on Model 1, further adjusted for total energy intake. Model 3, based on Model 2, further adjusted for phosphorus intake. * *p* < 0.05, ** *p* < 0.01, *** *p* < 0.001.

**Table 4:** Associations between protein in dairy product intake and the risk of fracture.

	No Consumption	0.1–2.8 g/Day	>2.8 g/Day
	(*n* = 11,777)	(*n* = 1956)	(*n* = 969)
Overall Participants			
Incidence (no. of fractures/ 1000 person-years)	3.747	1.808	3.419
Unadjusted Model	1.00 (Reference)	0.47 (0.34, 0.66) ***	0.65 (0.37, 1.13)
Model 1	1.00 (Reference)	0.37 (0.26, 0.53) ***	0.37 (0.20, 0.68) **
Model 2	1.00 (Reference)	0.38 (0.27, 0.54) ***	0.37 (0.20, 0.69) **
Model 3	1.00 (Reference)	0.38 (0.26, 0.54) ***	0.37 (0.20, 0.69) **
Men			
Incidence (no. of fractures/ 1000 person-years)	3.740	0.857	1.606
Unadjusted Model	1.00 (Reference)	0.22 (0.11, 0.45) ***	0.45 (0.17, 1.20)
Model 1	1.00 (Reference)	0.19 (0.09, 0.40) ***	0.33 (0.12, 0.92) *
Model 2	1.00 (Reference)	0.20 (0.10, 0.41) ***	0.33 (0.12, 0.92) *
Model 3	1.00 (Reference)	0.20 (0.10, 0.41) ***	0.34 (0.12, 0.96) *
Women			
Incidence (no. of fractures/ 1000 person-years)	3.753	2.606	2.712
Unadjusted Model	1.00 (Reference)	0.69 (0.47, 1.01)	0.82 (0.42, 1.61)
Model 1	1.00 (Reference)	0.52 (0.35, 0.80) **	0.39 (0.18, 0.85) *
Model 2	1.00 (Reference)	0.54 (0.35, 0.81) **	0.39 (0.18, 0.85) *
Model 3	1.00 (Reference)	0.53 (0.35, 0.81) **	0.38 (0.17, 0.83) *

Model 1 adjusted for age, sex, educational level, region of residence, individual annual income, smoking history, drinking history, BMI, chronic disease history, and physical activity. Model 2, based on Model 1, further adjusted for total energy intake. Model 3, based on Model 2, further adjusted for intakes of calcium and phosphorus. * *p* < 0.05, ** *p* < 0.01, *** *p* < 0.001.

## Data Availability

Publicly available datasets were analyzed in this study. This data can be found here: https://www.cpc.unc.edu/projects/china (accessed on 10 February 2022).

## Supplementary Materials

The following are available online at https://www.mdpi.com/article/10.3390/nu14081632/s1, Figure S1: Mediation effect of height and BMI on the association of dairy product intake with the risk of fracture compared to no consumption among subgroups among (a) men with dairy product intake of 0.1–100 g/day, (b) men with dairy product intake of >100 g/day, (c) women with dairy product intake of 0.1–100 g/day, (d) women with dairy product intake of >100 g/day, (e) women aged ≥ 45 years with dairy product intake of 0.1–100 g/day, and (f) women aged ≥ 45 years with dairy product intake of >100 g/day.
