# Supplementary material for: Association between Dairy Product Intake and Risk of Fracture among Adults: A Cohort Study from China Health and Nutrition Survey
Source: Nutrients. 2022 Apr 14;14(8):1632. doi: 10.3390/nu14081632 (PMC9027602; doi:10.3390/nu14081632)
Supplement: Supplementary file 1 [file nutrients-14-01632-s001.zip › nutrients-1625948-supplementary.pdf]

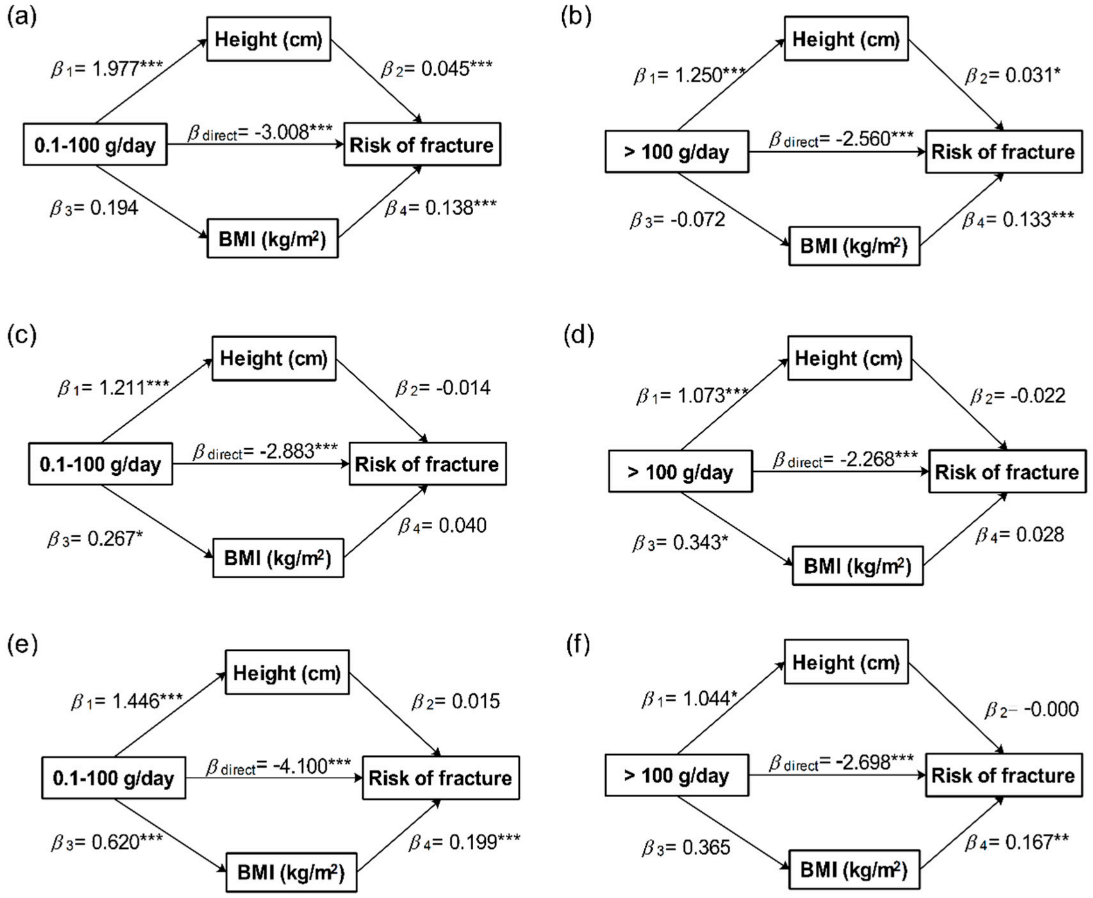

Supplementary Figure S1. Mediation effect of height and BMI on the association of dairy product intake with the risk of fracture compared to no consumption among subgroups among (a) Men with dairy product intake of 0.1-100 g/day, (b) Men with dairy product intake of >100 g/day, (c) Women with dairy product intake of 0.1-100 g/day, (d) Women with dairy product intake of >100 g/day, (e) Women aged ≥ 45 years with dairy product intake of 0.1-100 g/day, and (f) Women aged ≥ 45 years with dairy product intake of >100 g/day. Data are regression coefficients with adjustment for covariates (including age, educational level, city of residence, individual annual income, smoking history, drinking history, chronic disease history, physical activity, intake of energy and total calcium). *P<0.05, **P<0.01, ***P<0.001
